# Bilateral Multifocal Nodular Oncocytic Hyperplasia of the Parotid Gland Mimicking Warthin Tumor and Acinic Cell Carcinoma: A Case Report and Literature Review

**DOI:** 10.4317/jced.64152

**Published:** 2026-06-29

**Authors:** Martín Andura-Correas, Elena Ruiz-Bravo-Burguillos, Guillermo Chacón-Ferrer, Emilia María Montoro-Serrano, Javier González-Martín-Moro, María José Morán-Soto, José Luis Cebrián-Carretero

**Affiliations:** 1 Oral and Maxillofacial Surgery Department and Pathological Anatomy Department, Hospital Universitario La Paz, Madrid, Spain, Paseo De La Castellana, 261, Madrid

## Abstract

**Background:**

Multifocal nodular oncocytic hyperplasia (MNOH) of the salivary glands is a rare benign condition that can mimic both benign and malignant neoplasms, posing significant diagnostic challenges. Its bilateral and multifocal presentation is exceptionally uncommon. We present a unique case initially interpreted as bilateral Warthin tumor, later redefined as bilateral acinic cell carcinoma and finally reclassified as MNOH after comprehensive histopathological and immunohistochemical evaluation, and review the literature.

**Case Presentation:**

A 71-year-old woman presented with a long-standing mass in the left mandibular angle region. Fine-needle aspiration was compatible with Warthin tumor. MRI revealed a well-defined, partially cystic, enhancing lesion in the left parotid gland and a similar nodule in the contralateral gland. A left parotid tumorectomy with synchronous submandibular nodule excision was performed, both initially reported as acinic cell carcinoma (DOG1 positive). Subsequent left total parotidectomy showed multifocal acinic cell carcinoma, with negative FISH for CRTC1/MAML2, ruling out mucoacinar carcinoma. Contralateral MRI revealed multiple nodules with perfusion curves suggestive of Warthin tumor. Right parotidectomy revealed multifocal oncocytic hyperplasia with oncocytomas and extensive clear cell change. Immunohistochemistry demonstrated DOG1 apical positivity, but focal p63 expression and SOX10 negativity favored oncocytic hyperplasia/oncocytoma. Clinicopathologic correlation confirmed the diagnosis of bilateral MNOH.

**Discussion:**

This case illustrates the pitfalls in diagnosing oncocytic lesions, where radiology, cytology, and even immunohistochemistry may mimic acinic cell carcinoma. The review of published cases confirms the rarity of bilateral MNOH and highlights the importance of correlating morphology, immunoprofiles, and clinical context.

**Conclusions:**

MNOH should be considered in the differential diagnosis of bilateral parotid lesions to avoid overtreatment. Recognition of its histological spectrum, including clear cell and oncocytoma-like features, is essential for accurate diagnosis.

## Introduction

Oncocytic lesions of the salivary glands are rare, representing less than 1% of salivary tumors (1). According to the World Health Organization (WHO), they include oncocytosis, oncocytoma, and oncocytic carcinoma. Multifocal nodular oncocytic hyperplasia (2 - 10) (MNOH), also termed adenomatous oncocytic hyperplasia, is a benign, non-neoplastic process composed of multiple oncocytic nodules. Its clinical and radiological features overlap with benign and malignant tumors such as Warthin tumor (2 , 3 , 8), oncocytoma, and acinic cell carcinoma (13 - 15), making diagnosis challenging. We present a case of bilateral MNOH initially misdiagnosed as Warthin tumor and later as synchronous multifocal acinic cell carcinoma, and provide a review of the literature.

## Case Report

**Figure 1:**
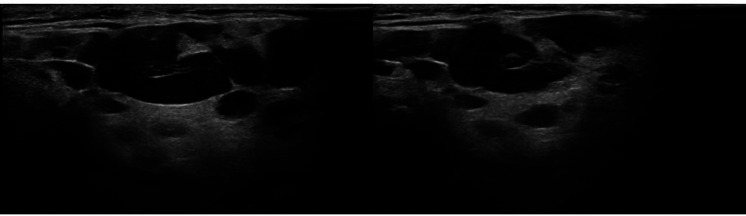
Ultrasound examination shows normal-sized bilateral parotid glands containing multiple bilateral solid hypoechoic nodular lesions.

A 71-year-old woman with no relevant personal and family medical history was referred to the Oral and Maxillofacial Surgery outpatient clinic due to a slowly enlarging mass in the left mandibular angle region, first noticed several years earlier. The swelling was painless, firm, and mobile over deeper planes, with no associated facial nerve dysfunction or cervical lymphadenopathy. The patient reported no xerostomia, dysphagia, or systemic symptoms. Her medical history was unremarkable for autoimmune or neoplastic diseases. Ultrasound examination performed during ultrasound-guided fine-needle aspiration (US-guided FNA) showing normal-sized bilateral parotid glands containing multiple bilateral solid hypoechoic nodular lesions (Fig. 1).

1

**Figure 2:**
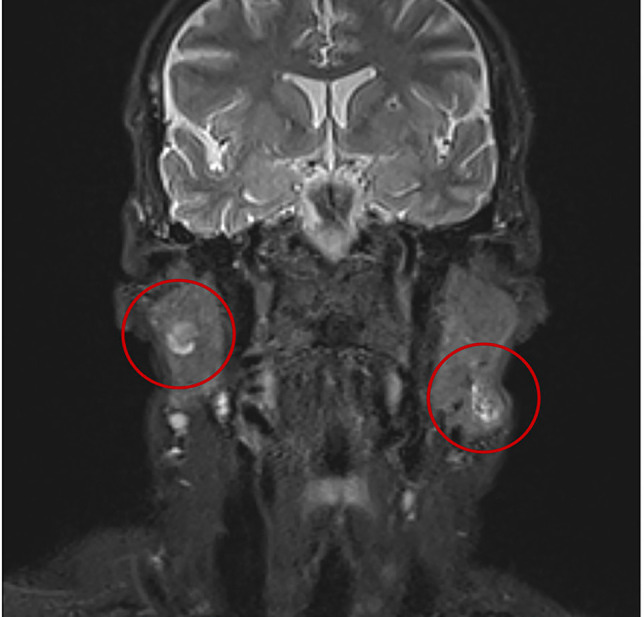
Initial magnetic resonance imaging (MRI) in T2-weighted STIR 3D sequence showing multiple bilateral parotid nodules, appearing as hyperintense lesions within both parotid glands.

Fine-needle aspiration (FNA) cytology of the lesion revealed oncocytic epithelial cells with granular cytoplasm and lymphoid stroma, leading to an initial interpretation consistent with Warthin tumor (2 , 3 , 8). Given the chronicity of the lesion and the cytological impression, magnetic resonance imaging (MRI) was requested for further characterization. MRI demonstrated a well-circumscribed nodule located in the tail of the left parotid gland measuring 15 × 5 millimeters (mm) on axial and anteroposterior planes. The lesion appeared hypointense on T2-weighted sequences, with partially cystic features and moderate enhancement after contrast administration. These findings were considered compatible with Warthin tumor (2 , 3 , 8). In addition, the imaging study revealed another smaller nodule of similar characteristics, measuring 12 mm, in the superficial lobe of the right parotid gland. No cervical lymphadenopathy of pathological size was noted (Fig. 2).

2

**Figure 3:**
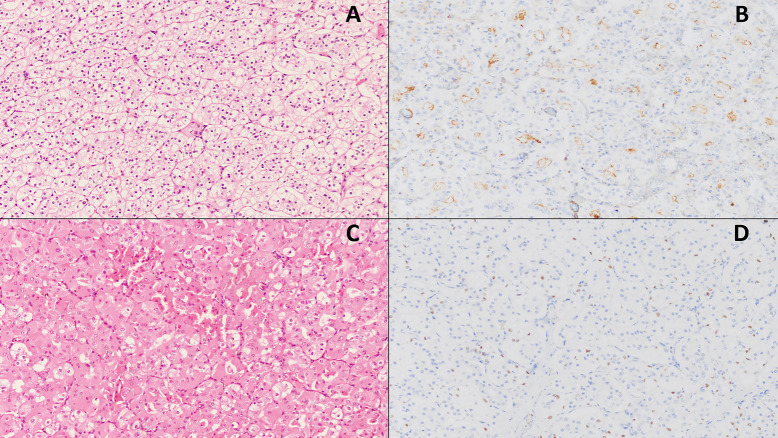
Inmunohistological findings. A: The tumor shows an acinar growth pattern, with formation of small or poorly defined glandular lumina. Tumor cells display abundant clear cytoplasm and small nuclei without nuclear atypia. B: Immunohistochemical staining for DOG1 demonstrates apical membranous positivity in tumor cells. C: Within the tumor specimen, a tumor nodule composed of oncocytic cells is identified. D: Immunohistochemical staining for p63 shows scattered positivity within the tumor cell population.

Based on these findings, the working diagnosis was bilateral Warthin tumor (2 , 3 , 8). Surgical management was planned, and the patient underwent tumorectomy of the left parotid lesion together with excision of an independent left submandibular nodule identified intraoperatively. Histopathological evaluation of both specimens, however, contradicted the initial diagnosis. The lesions were reported as acinic cell carcinoma (13 - 15), characterized by serous acinar differentiation, absence of perineural or vascular invasion, and tumor reaching surgical margins (Fig. 3A).

3

Immunohistochemistry demonstrated DOG1 (15 , 16) positivity with an apical membranous staining pattern, supporting the diagnosis of acinic cell carcinoma (Fig. 3B) (13 - 15). This unexpected finding significantly altered the diagnostic and therapeutic considerations. In light of the malignant diagnosis, a total parotidectomy of the left gland was subsequently performed. Histological evaluation of the surgical specimen revealed multifocal lesions consistent with acinic cell carcinoma (13 - 15). Given recent descriptions of the so-called mucoacinar carcinoma (a morphologic variant of mucoepidermoid carcinoma with acinar differentiation associated with CRTC1/MAML2 translocation) (14), fluorescence in situ hybridization (FISH) was performed. The analysis was negative for CRTC1/MAML2 (14), excluding this entity. The cumulative findings continued to suggest multifocal acinic cell carcinoma (13 - 15) of the left parotid. During follow-up, MRI of the contralateral parotid was performed. The study showed multiple solid nodules distributed throughout the superficial lobe of the right parotid, appearing hypointense on T1-weighted and isointense on T2-weighted sequences. Compared with normal parotid parenchyma, these nodules showed lower enhancement. Perfusion studies demonstrated early uptake and washout, a curve pattern classically described for Warthin tumor (2 , 3 , 8). Given the multiplicity of lesions and the patient's history, surgical removal of the right parotid was indicated. A total right parotidectomy was carried out. Histopathological examination revealed multifocal oncocytic nodules, oncocytomas, and extensive areas of clear cell changes. Morphology showed remarkable similarity to the previously resected left parotid lesions, although in this specimen a distinct oncocytic nodule suggestive of oncocytic hyperplasia was clearly identified (Fig. 3C). These findings prompted a thorough reevaluation of the entire case in correlation with the clinical and radiological features. Additional immunohistochemical studies provided further insight. Although DOG1 (15 , 16) staining was apical, moderate-to-strong, and present in more than 50% of the cells-criteria that in many settings would strongly support a diagnosis of acinic cell carcinoma (13 - 15)-the tumor also demonstrated focal p63 (16) expression and was negative for SOX10 (Fig. 3D) (17). This immunoprofile is more consistent with oncocytic hyperplasia or oncocytoma rather than true acinic cell carcinoma (13 - 15). The combination of bilaterality, multifocality, oncocytic morphology, and discordant immunohistochemical features supported a benign diagnosis. After multidisciplinary discussion and review of the histological slides, the final diagnosis was revised to bilateral multifocal nodular oncocytic hyperplasia (2 - 10) (MNOH) of the parotid glands, with associated oncocytomas and clear cell changes. The patient's postoperative recovery was uneventful. No recurrence has been detected during follow-up clinical and radiological evaluations. She remains disease-free to date, with normal facial nerve function and good quality of life.

## Discussion

The present case highlights the exceptional diagnostic challenges encountered when dealing with oncocytic lesions of the salivary glands, and illustrates the complex interplay between clinical findings, radiological interpretation, cytological impressions, histological assessment, and immunohistochemical as well as molecular studies. In this discussion we aim to contextualize the case within the broader literature on multifocal nodular oncocytic hyperplasia (MNOH), emphasizing diagnostic pitfalls, differential diagnoses, therapeutic implications, and lessons for clinical practice. Oncocytic lesions of the salivary glands are rare, constituting less than 1% of all salivary gland tumors (1). They comprise a spectrum of entities including oncocytosis (diffuse or multifocal hyperplasia of oncocytic cells), oncocytoma (a benign neoplasm), and oncocytic carcinoma (a malignant neoplasm) (9). MNOH, also referred to as adenomatous oncocytic hyperplasia, is a non-neoplastic process in which oncocytic cells proliferate in discrete nodules within the salivary parenchyma. This process may be unilateral or bilateral, solitary or multifocal, and is typically discovered incidentally or in the workup of parotid gland masses. The rarity of MNOH is compounded by its ability to mimic both benign and malignant salivary tumors clinically and radiologically. In our patient, the initial presentation with a long-standing parotid mass, the FNA cytology showing oncocytic cells with lymphoid background, and the MRI features of a partially cystic, enhancing nodule in the parotid tail were all consistent with a preliminary diagnosis of Warthin tumor (2 , 3 , 8). The identification of a contralateral lesion with similar features further reinforced this impression. However, the subsequent histological interpretation as acinic cell carcinoma dramatically changed the clinical trajectory, leading to more aggressive surgery and molecular testing (13 - 15). This sequence of events exemplifies how MNOH can masquerade as diverse pathologies and mislead both pathologists and surgeons. Radiological considerations are central to the diagnostic challenge. MRI features of MNOH are nonspecific, often overlapping with Warthin tumor and oncocytoma (8). In our case, the contralateral parotid lesions exhibited perfusion curves with early uptake and washout, a hallmark of Warthin tumor. Several authors have reported similar observations, underscoring the limited discriminative power of imaging alone in distinguishing these entities. While diffusion-weighted imaging and dynamic contrast-enhanced studies can provide additional clues, they are not definitive. The multifocal and bilateral distribution, however, should always raise suspicion for oncocytosis or MNOH rather than discrete neoplasms (2 , 3 , 8). Cytology, although minimally invasive, is similarly limited (3). Fine-needle aspiration often reveals oncocytic cells with abundant granular cytoplasm, prominent nucleoli, and sometimes lymphoid stroma, findings that are equally compatible with Warthin tumor, oncocytoma, or even oncocytic carcinoma (7). Misinterpretations are frequent. In our patient, the preoperative FNA was interpreted as Warthin tumor, consistent with the radiological impression but discordant with the eventual histological findings. This underscores the fact that cytology cannot reliably distinguish between the spectrum of oncocytic lesions. Histologically, MNOH can simulate acinic cell carcinoma, as vividly illustrated in our case. Acinic cell carcinoma is characterized by serous acinar differentiation, with cells showing basophilic granular cytoplasm due to zymogen granules. However, oncocytic cells with abundant mitochondria may exhibit overlapping morphology. Moreover, multifocality within a single gland may be misinterpreted as multifocal carcinoma rather than hyperplasia. In our patient, the left parotidectomy specimen was interpreted as multifocal acinic cell carcinoma, leading to further concern for bilateral malignant disease. This interpretation highlights the need for caution in diagnosing carcinoma in the absence of clear invasive features, particularly when the lesions are bilateral and multifocal (13 - 15). Immunohistochemistry provides useful markers but must be interpreted with context. DOG1 (Discovered On GIST-1) has been widely used as a marker for acinic cell carcinoma, typically showing apical membranous staining in tumor cells. In our patient, DOG1 was positive in more than 50% of cells with a strong apical pattern, which strongly supported acinic cell carcinoma at first glance. However, recent literature has cautioned that DOG1 positivity is not entirely specific and can also be observed in oncocytic lesions. Additional markers, such as SOX10 and p63, are helpful in this regard. In our case, SOX10 negativity and focal p63 expression were more consistent with oncocytic hyperplasia or oncocytoma than with true acinic cell carcinoma. This immunohistochemical discrepancy was critical in prompting re-evaluation of the diagnosis (15 , 16). Molecular testing has introduced new dimensions to the differential diagnosis. The recently described mucoacinar carcinoma (also called acinic-like mucoepidermoid carcinoma) shares many histological features with acinic cell carcinoma but is distinguished by the presence of the CRTC1/MAML2 fusion, a hallmark of mucoepidermoid carcinoma. In our patient, FISH analysis for CRTC1/MAML2 was negative, excluding mucoacinar carcinoma (14). Although this did not resolve the diagnostic dilemma, it eliminated one potential mimic and underscored the importance of molecular analysis in challenging cases. The ultimate resolution of the case rested on clinicopathological correlation. The bilateral and multifocal distribution of lesions, the identification of a distinct oncocytic nodule in the right parotidectomy specimen, the immunohistochemical profile with DOG1 positivity but SOX10 negativity, and the absence of aggressive histological features all converged to support a diagnosis of MNOH rather than carcinoma. This illustrates the importance of integrating clinical context with pathological findings, especially in rare entities with overlapping features (17). From a therapeutic standpoint, the implications are profound. Acinic cell carcinoma is a malignant tumor that generally requires total parotidectomy and sometimes adjuvant therapy. In contrast, MNOH is a benign process that may not require surgical intervention beyond diagnostic excision. In our patient, the misdiagnosis led to bilateral total parotidectomies, procedures associated with risks such as facial nerve injury, Frey syndrome, and cosmetic deformity. Although the patient recovered uneventfully, the case underscores the potential for overtreatment when MNOH is mistaken for carcinoma. Awareness of this entity among surgeons and pathologists is crucial to avoid unnecessary morbidity. The literature on MNOH is limited but growing. A review of reported cases (Table 1) indicates that most patients are middle-aged to elderly, with no clear gender predilection.

Table 1

The parotid glands are most commonly affected, although submandibular involvement has been documented. Bilateral cases remain exceptional, with only a handful of reports in the literature. Variants including cystadenomatous, clear cell, and oncocytoma-like changes have been described, echoing the findings in our patient. Misdiagnosis as Warthin tumor or acinic cell carcinoma has been reported by multiple authors, reinforcing the diagnostic dilemma posed by MNOH (2 - 9). In comparing our case with published reports, several unique aspects stand out. First, the sequence of misinterpretations spanning cytology, imaging, histology, immunohistochemistry, and even molecular studies highlights the multilayered nature of the diagnostic challenge. Second, the coexistence of oncocytomas and clear cell changes within the hyperplastic process adds further complexity and expands the recognized histological spectrum of MNOH. Third, the necessity for bilateral total parotidectomy illustrates the clinical consequences of misdiagnosis, making this case an instructive cautionary tale. The lessons learned from this case are several. For clinicians, bilateral and multifocal parotid lesions should always prompt consideration of oncocytic hyperplasia, even in the presence of atypical histological features. For pathologists, reliance on a single immunohistochemical marker such as DOG1 is insufficient; a panel including SOX10, p63, and others should be employed. For both, close collaboration and correlation of clinical, radiological, and pathological data are essential. Finally, the reporting of additional cases will help refine diagnostic criteria, raise awareness, and potentially reduce unnecessary radical surgery in future patients. In conclusion, MNOH is a rare but important mimic of both benign and malignant salivary gland tumors. The present case, initially misdiagnosed as bilateral multifocal acinic cell carcinoma, was ultimately recognized as bilateral MNOH after comprehensive re-evaluation. This case underscores the importance of awareness, multidisciplinary collaboration, and caution in the interpretation of oncocytic salivary lesions. Through greater recognition of its clinicopathological spectrum, clinicians and pathologists can avoid misdiagnosis and the potential for overtreatment.

## Conclusions

Multifocal nodular oncocytic hyperplasia is a rare benign process that may closely mimic acinic cell carcinoma and other oncocytic tumors. Bilateral presentation is exceptionally uncommon. Recognition of its spectrum of histological patterns, together with appropriate use of immunohistochemical markers and correlation with clinical presentation, is crucial to prevent misdiagnosis and overtreatment.

## Figures and Tables

**Table 1:** Previously published cases of multifocal nodular oncocytic hyperplasia of the regional and salivary glands.

Author (Year)	Age/Sex	Gland(s)	Laterality	Histological Variant	Management
Houas et al. (2024)	52/F	Parotid	Bilateral	Classic	Parotidectomy
Hammami et al. (2021)	43/F	Parotid	Bilateral	Clear cell + oncocytoma	Parotidectomy
Zhu et al. (2021)	Mother & son	Parotid	Bilateral (familial)	Classic	Parotidectomy
Bannister et al. (2018)	57/M	Submandibular	Bilateral (non-synchronous)	Papillary cystadenoma-like	Bilateral excision
Albader et al. (2023)	73/F	Parotid	Unilateral	Cystadenomatous	Superficial parotidectomy
Kinoshita et al. (2014)	71/F	Parotid	Unilateral	Classic	Parotidectomy
Yue et al. (2021)	66/M	Parotid	Unilateral (recurrent)	Cystic oncocytosis	Revision parotidectomy
Topalidis et al. (2024)	40s/F	Thyroid	Unilateral	Oncocytic hyperplastic nodule	Thyroidectomy

1
